# LncRNA FOXC2-AS1 enhances FOXC2 mRNA stability to promote colorectal cancer progression via activation of Ca^2+^-FAK signal pathway

**DOI:** 10.1038/s41419-020-2633-7

**Published:** 2020-06-08

**Authors:** Ke Pan, Yong Xie

**Affiliations:** Department of General Surgery, The Second Xiangya Hospital of Central South University, Changsha, Hunan China

**Keywords:** Gastrointestinal cancer, Prognostic markers, Colorectal cancer

## Abstract

Long noncoding RNAs (lncRNAs) have been confirmed, which are involved in tumorigenesis and metastasis in colorectal cancer (CRC). FOXC2 antisense RNA 1 (FOXC2-AS1) was reported, facilitating the proliferation and progression in several cancers. However, the role of FOXC2-AS1 in CRC cell migration and metastasis is not unclear. In this study, we observed that lncRNA FOXC2-AS1 was upregulated in CRC tissues, and its high expression indicated the poor survival in CRC patients. Meanwhile, FOXC2-AS1 was higher in CRC tissues with metastasis than that of nonmetastatic tumor tissues. We found that FOXC2-AS1 was predominately expressed in the nucleus of tissues and cells. FOXC2-AS1 knockdown suppressed CRC cell growth, invasion, and metastasis in vitro and in vivo. Moreover, FOXC2-AS1 could positively regulate the neighboring gene FOXC2 and stabilized FOXC2 mRNA by forming a RNA duplex. Meanwhile, ectopic expression of FOXC2 could obviously alleviate the suppressed effects caused by silencing FOXC2-AS1. For the mechanism, FOXC2-AS1 knockdown could reduce intracellular Ca^2+^ levels, inhibited FA formation and FAK signaling, and these suppressed effects were mitigated by increasing FOXC2 expression. These results demonstrated that FOXC2-AS1 enhances FOXC2 mRNA stability to promote CRC proliferation, migration, and invasion by activation of Ca^2+^-FAK signaling, which implicates that FOXC2-AS1 may represent a latent effective therapeutic target for CRC progression.

## Introduction

Colorectal cancer (CRC) is a common digestive tract malignancy, accounting for about 40% new tumors of the digestive tract. According to the survey, there were nearly 1.3 million new CRC cases and more than 0.6 million deaths worldwide every year^1^. In recent years, remarkable progress has been made in CRC treatment, but over half of the patients with advanced CRC die from recurrence and metastasis, which led to the unfavorable prognosis^2,3^. Thus, a better illustration of the molecular mechanisms regarding CRC progression is urgently needed.

Long noncoding RNAs (lncRNAs) are a kind of RNA transcripts with lengths >200 nt, and limit or lack protein-coding capacity^4^. LncRNAs demonstrated widespread expression in animals and plants, which can exhibit function in *cis*-regulated expression of neighboring genes or in *trans*-regulation distal genes^5^. Numerous literature has confirmed that the dysregulation of lncRNAs is involved in the pathophysiology of numerous human disorders, especially in cancer^6,7^. lncRNA H19, MALAT1, HOTAIR, etc., were widely reported to be involved in human tumorigenesis and metastasis^8–10^. LncRNA LNMAT1 promotes bladder cancer metastasis through enhancing CCL2- dependent macrophage recruitment^11^; LncRNA MFI2-AS1 facilitates cell migration and invasion via acting as the ceRNA of miR-574 to regulate MYCBP in CRC^12^; HOXD-AS1 represses CRC growth and metastasis through suppressing HOXD3-activated integrin β3 transcription and MAPK/AKT pathway^13^. Thus, lncRNAs may play a key role in cancer metastastis; exploring the molecular mechanisms will be significant for cancer therapy.

FOXC2-AS1 is an antisense RNA transcript of forkhead box protein C2 (FOXC2). Research has shown that FOXC2-AS1 was highly expressed in breast cancer, prostate cancer, and non-small- cell lung cancer (NSCLC); the upregulated expression of FOXC2-AS1 predicted poor survival, and facilitated proliferation and progression of these carcinomas^14–16^. Besides, FOXC2-AS1 was reported to promote doxorubicin resistance in osteosarcoma^17^. Yet, FOXC2-AS1 is not well-characterized in cancers, including CRC. The expression, function, and mechanism of FOXC2-AS1 has not been reported in CRC.

In this research, we found that FOXC2-AS1 was obviously upregulated in metastasis CRC tissue, and relates to the clinicopathologic features and poor prognosis in CRC patients. FOXC2-AS1 depletion significantly weakened CRC cell growth, invasion, and metastasis in vitro and in vivo. Moreover, FOXC2-AS1 could promote FOXC2 expression via enhancing its mRNA stability. Further investigation demonstrated that FOXC2-AS1 could increase intracellular Ca^2+^ levels and promote FA formation via activation of Ca^2+^-FAK signaling pathway, ultimately contributing to CRC proliferation, migration, and invasion. Our investigation revealed the oncogenic role of FOXC2-AS1 through increased intracellular Ca^2+^ levels and promoted FA formation in CRC, and implied FOXC2-AS1 as a latent prognostic marker and therapeutic target for CRC.

## Results

### FOXC2-AS1 is upregulated in CRC tissues and predicts poor prognosis

**Fig. 1:**
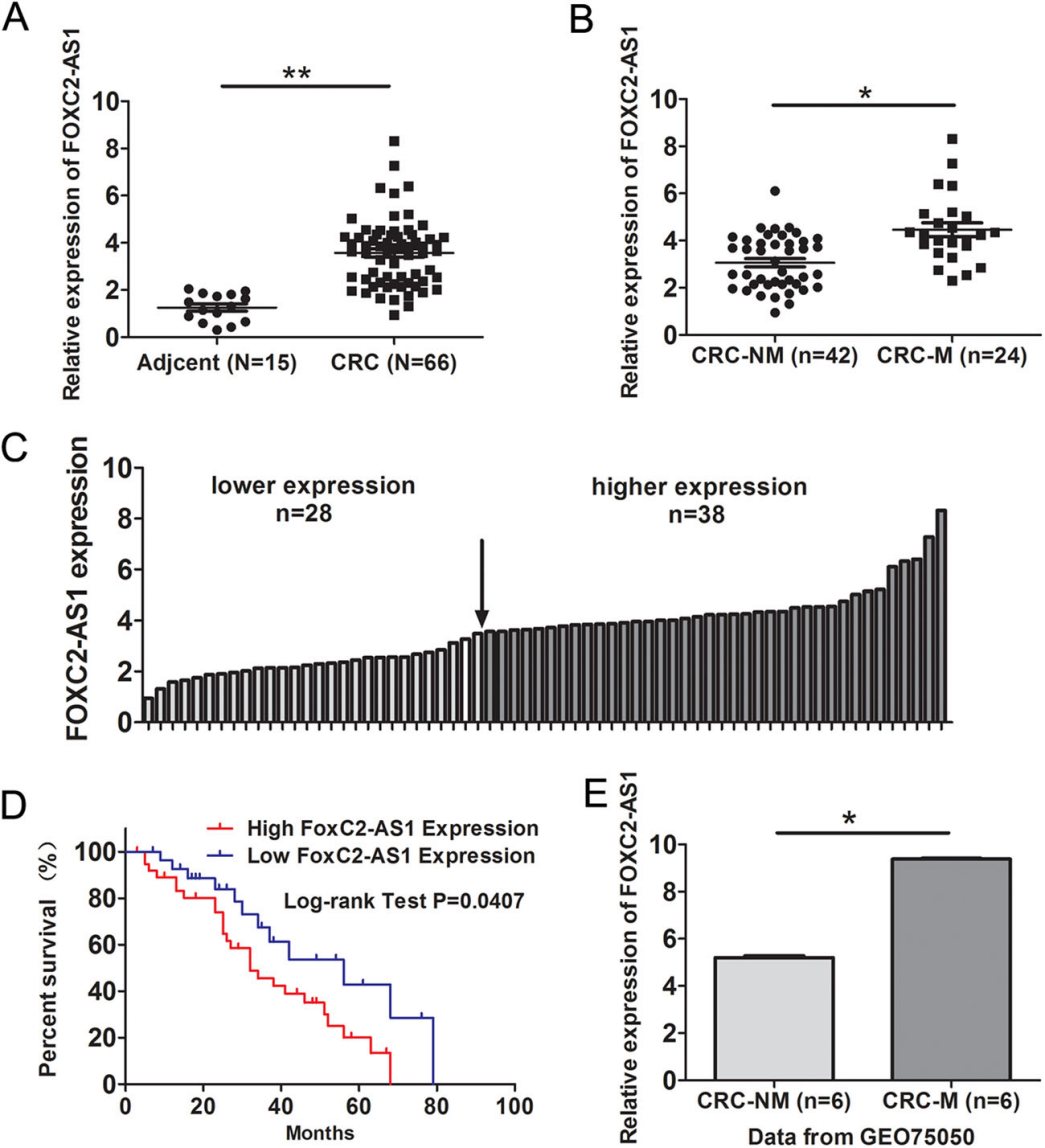
FOXC2-AS1 is upregulated in CRC tissues and predicts poor prognosis. **a** FOXC2-AS1 expression was examined in 66 CRC and 15 adjacent normal tissues by qRT-PCR method. **b** FOXC2-AS1 expression in CRC with metastasis and nonmetastatic tumor tissues. **c** FOXC2-AS1 expression was classified into high and low groups according to the median expression level in CRC tissues. **d** The overall survival of CRC patients was assessed using Kaplan–Meier analysis. **f** FOXC2-AS1 expression in GEO data (GSE75050). *P < 0.05, **P < 0.01.

In 66 CRC and 15 adjacent normal tissues, FOXC2-AS1 expression was elevated in CRC tissues by qRT-PCR assay (Fig. 1a). Moreover, FOXC2-AS1 had a higher expression in CRC tissues with metastasis than nonmetastatic tumor tissues (Fig. 1b). Further, based on the median FOXC2-AS1 expression in tumor tissues, 66 CRC patients were classified into high group (*n* = 38) and low group (*n* = 28) (Fig. 1c). The association between FOXC2-AS1 expression and clinical pathology parameters was analyzed employing the χ^2^ test. As shown in Table 1, the high expression of FOXC2-AS1 was significantly correlated with TNM stage and metastasis. Kaplan–Meier analysis showed that patients with high FOXC2-AS1 expression had a significantly poorer overall survival than those with low FOXC2-AS1 expression (Fig. 1d). Besides, our result was consistent with the GEO data (GSE75050), which found that FOXC2-AS1 was consistently upregulated in CRC tissue with liver metastasis compared with CRC tissue without metastasis (Fig. 1e). These data indicated that FOXC2-AS1 may be an oncogene in CRC and has an impact on patient survival.

**Table 1:** The association between FOXC2-AS1 expression and clinical pathology features.

Variable	Cases	FoxC2-AS1 expression		*P* value	χ^2^ Value
		Low	High		
Age				0.438	0.844
<60	23	8	15		
≥60	43	20	23		
Gender				0.797	0.179
Male	42	17	25		
Female	24	11	13		
Tumor location				0.621	0.319
Rectum	38	15	23		
Colon	28	13	15		
Tumor size				0.609	0.512
<5	25	12	13		
≥5	41	16	25		
Lymphovascular invasion				0.451	0.68
Negative	41	19	22		
Positive	25	9	16		
TNM				0.042	5.092
I+II	39	21	18		
III+IV	27	7	20		
Distant metastasis				0.04	4.688
No	42	22	20		
Yes	24	6	18		

### FOXC2-AS1 depletion suppresses CRC cell growth, invasion, and metastasis in vitro and in vivo

**Fig. 2:**
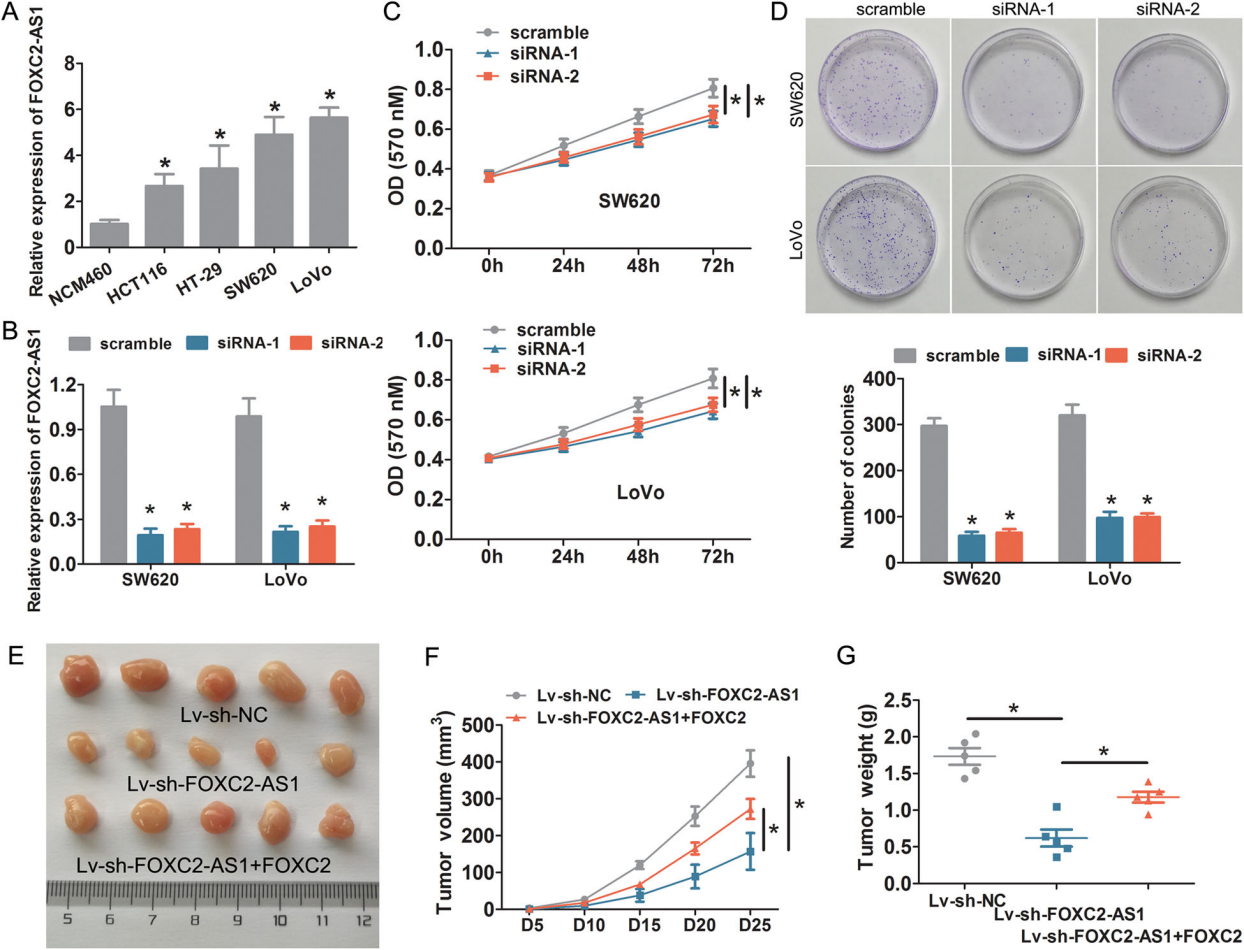
Knockdown of FOXC2-AS1 suppresses CRC cell growth and proliferation in vitro and in vivo. **a** The endogenous expression of FOXC2-AS1 was examined in CRC cell lines (HCT116, HT-29, SW620, and LoVo) and normal colonic cell line NCM460. **b** Knockdown efficiency was examined by qRT-PCR in SW620 and LoVo cells. MTT (**c**) and clone-formation assays (**d**) were used to examine the effect of FOXC2-AS1 depletion on CRC cell proliferation and growth. **e** Representative images of xenograft tumors produced by FOXC2-AS1-silenced LoVo cells or control cells in nude mice. The effect of FOXC2-AS1 knockdown on tumor growth (**f**) and weight (**g**) in in vivo tumor xenograft experiments. *P < 0.05.

**Fig. 3:**
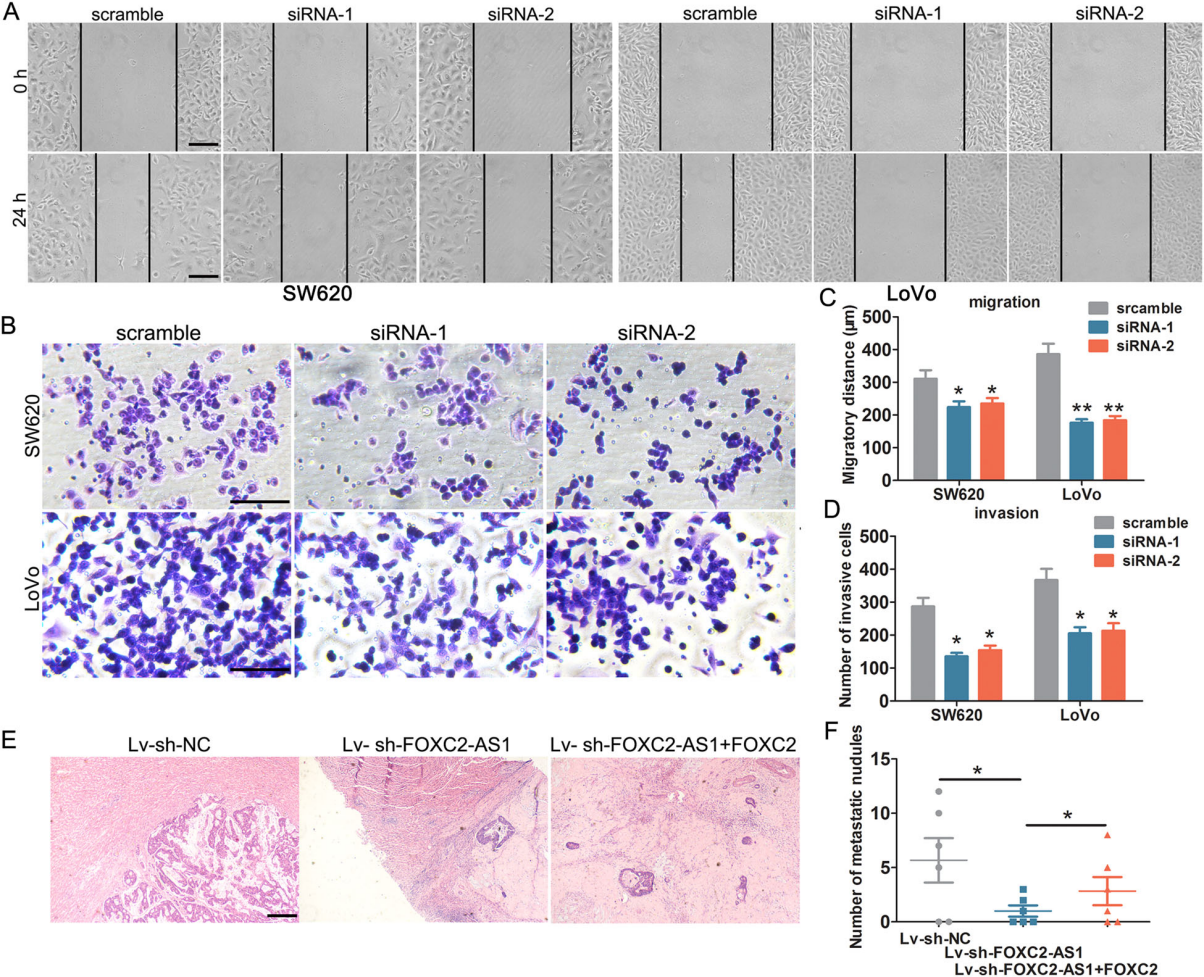
FOXC2-AS1 depletion impedes CRC cell invasion and metastasis in vitro and in vivo. FOXC2-AS1 knockdown significantly suppressed CRC cell migration and invasion, as detected by wound healing (**a**) and Transwell assay (**b**). The data statistics of wound-healing assay (**c**) and Transwell assay. **d** The effect of FOXC2-AS1 on CRC liver metastasis in vivo, the metastatic nodules in liver tissue were detected by HE staining (**e**), and data statistics of metastatic nodules (**f**). Scale bars = 100 μm *P < 0.05, **P < 0.01.

We investigate the role of FOXC2-AS1 on CRC cells. First, we detected FOXC2-AS1 endogenous expression in CRC cell lines (HCT116, HT-29, SW620, and LoVo) and normal colonic cell line NCM460 by qRT-PCR. As shown in Fig. 2a, FOXC2-AS1 was significantly upregulated in these tumor cell lines compared with NCM460 cells. Especially, SW620 and LoVo cells had a higher expression than HCT116 and HT-29 cells. Therefore, SW620 and LoVo were chosen for loss-of-function assays. Experimental results confirmed that FOXC2-AS1 expression was effectively suppressed through transfection of FOXC2-AS1 siRNA-1 and siRNA-2 (Fig. 2b). MTT and clone-formation assays showed that FOXC2-AS1 depletion significantly impeded cell proliferation and growth (Fig. 2c, d). Notably, FOXC2-AS1 knockdown obviously suppressed the tumor volume, growth rates, and weight of the subcutaneous xenografts in nude mice (Fig. 2e–g). Next, wound healing and Transwell experiments were applied to explore the effects of FOXC2-AS1 on CRC cell migration and invasion. The results showed that FOXC2-AS1 knockdown obviously impaired CRC cell migration (Fig. 3a, b) and invasion (Fig. 3c, d). Besides, we investigated the effect of FOXC2-AS1 on CRC liver metastasis in vivo. As shown in Fig. 3e, f, the FOXC2-AS1-knockdown group displayed a reduced number of metastatic nodules in livers compared with the control group. Besides, in normal colonic cells of FHC, we observed that these phenotypes were also suppressed after FOXC2-AS1 silencing (Fig. S1). Our results indicated that FOXC2-AS1 could promote CRC cell growth, invasion, and metastasis in vitro and in vivo.

### FOXC2-AS1 regulated tumor behavior via enhancing the stability of FOXC2 mRNA

**Fig. 4:**
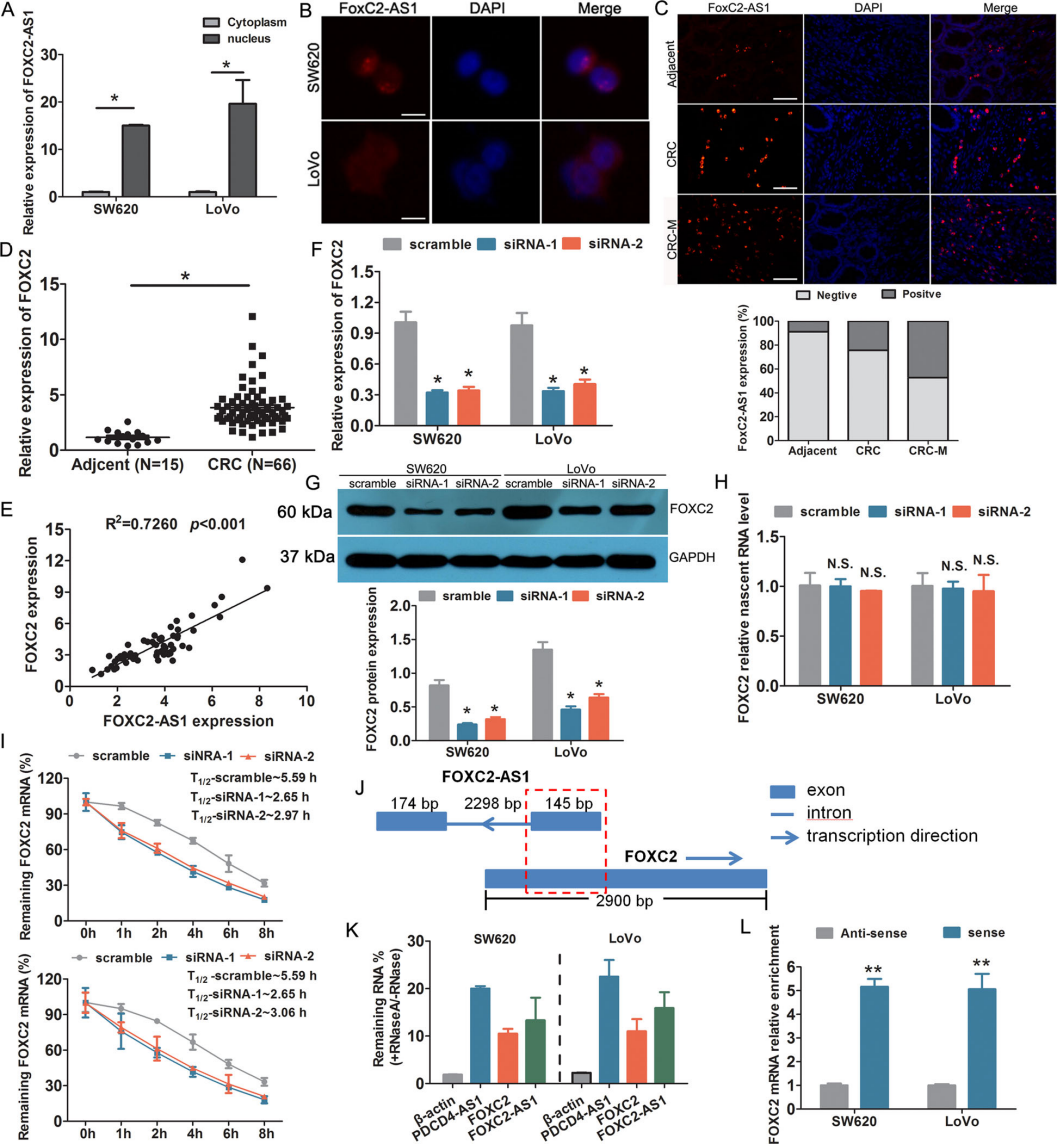
FOXC2-AS1 positively regulated FOXC2 expression via enhancing FOXC2 mRNA stability. **a** The relative expression of FOXC2-AS1 in the cytoplasm and nucleus of SW480 and LoVo cells. FISH detection was used to investigate the location of FOXC2-AS1 in cells (**b**) and tissues (**c**). Scale bars = 100 μm. **d** The expression of FOXC2 was examined in 66 CRC and 15 adjacent normal tissues. **e** The expression relationship between FOXC2-AS1 and FOXC2 was analyzed in 66 CRC tissues. FOXC2 expression was examined in FOXC2-AS1-silenced SW480 and LoVo cells by qRT-PCR method (**f**) and Western blot (**g**). **h** qRT-PCR detected the levels of nascent FOXC2 pre-mRNA with Click-iT Nascent RNA Capture Kit in FOXC2-AS1-silenced and control cells. **i** qRT-PCR investigated FOXC2 mRNA stability in FOXC2-AS1-silenced and control cells treated with the transcriptional inhibitor actinomycin D (50 ng/ml) for different times. **j** Schematic diagram of FOXC2-AS1 and FOXC2 gene locus and structure. The red grid represents the completely complementary region. The number represents the length of exon or intron. **k** qRT-PCR was conducted to analyze RNase protection experiment. β-actin was used as a negative control, while PDCD4-AS1 was used as a positive control. **l** qRT-PCR was performed to assess the interaction between FOXC2 and biotin-labeled FOXC2-AS1 after RNA pull-down assay. *P < 0.05, ***P < 0.001, N.S. indicates not statistically different.

Previous experiments have confirmed the function of FOXC2-AS1 in CRC, but the underlying mechanisms need to be investigated. Subcellular fractionation detection revealed that FOXC2-AS1 was predominately located in the nucleus of SW620 and LoVo cells (Fig. 4a). FISH analysis also confirmed the expression localization of FOXC2-AS1 in cells and tissues (Fig. 4b, c). Moreover, the percent of positive signal in CRC tissues was higher than adjacent tissues; meanwhile, CRC tissues with metastasis had a more higher signal than nonmetastatic tumor tissue. Evidence has confirmed that antisense lncRNAs could regulate the expression of their neighboring genes^18^, and FOXC2 was the nearest gene, having some overlap sequences with FOXC2-AS1. Thus, we investigated whether FOXC2-AS1 regulates FOXC2 expression. First, FOXC2 expression was detected in CRC tissues and cell lines. It is found that FOXC2 was elevated in CRC specimens, which was consistent with the expression of FOXC2-AS1 (Fig. 4d). Besides, we found a positive relevance between the expression of FOXC2 and FOXC2-AS1 in CRC tissues (Fig. 4e). Knockdown of FOXC2-AS1, FOXC2 mRNA, and protein levels was obviously downregulated compared with control cells (Fig. 4f, g). These results confirmed that FOXC2-AS1 can positively regulate FOXC2 expression.

Antisense RNA transcripts could regulate their sense genes in the transcriptional or post- transcriptional level^19^. To investigate whether FOXC2-AS1 regulates FOXC2 transcription, we examined the levels of nascent FOXC2 pre-mRNA with Click-iT Nascent RNA Capture Kit (Life Technologies, USA) in FOXC2-AS1-silenced and control cells. The results displayed that FOXC2-AS1 knockdown does not influence the level of nascent FOXC2 pre-mRNA (Fig. 4h), which implied that FOXC2-AS1 does not regulate FOXC2 expression at the transcriptional level. Next, we investigated whether FOXC2-AS1 modulated post-transcriptional process of FOXC2 mRNA by RNA-stability assay. FOXC2-AS1-silenced and control cells were treated with the transcriptional inhibitor actinomycin D (Sigma-Aldrich, USA) for different times; then cells were collected, and we conducted qRT-PCR to examine the level of FOXC2 mRNA. The result showed that the half-life of FOXC2 mRNA in FOXC2-AS1-silenced cells was decreased by almost half compared with scramble cells (Fig. 4i). It is suggested that FOXC2-AS1 could enhance its mRNA stability. Antisense lncRNAs could regulate sense mRNA stability by forming RNA duplexes^20,21^. Based on the locus and gene structure, exon 1 of FOXC2-AS1 was completely complementary with FOXC2, implying that FOXC2-AS1 and FOXC2 may form a RNA duplex (Fig. 4j). Following this, RNase protection experiment was conducted to test our hypothesis using single-strand-specific RNA-degrading RNase A. As shown in Fig. 4k, FOXC2-AS1 and FOXC2 mRNA were protected against RNase A cleaving, as PDCD4-AS1 was used as a positive control^20^; this result indicated that the RNA duplex existed between FOXC2-AS1 and FOXC2 mRNA. In addition, RNA pull-down assay confirmed the remarkable interaction between FOXC2-AS1 and FOXC2 (Fig. 4l).

**Fig. 5:**
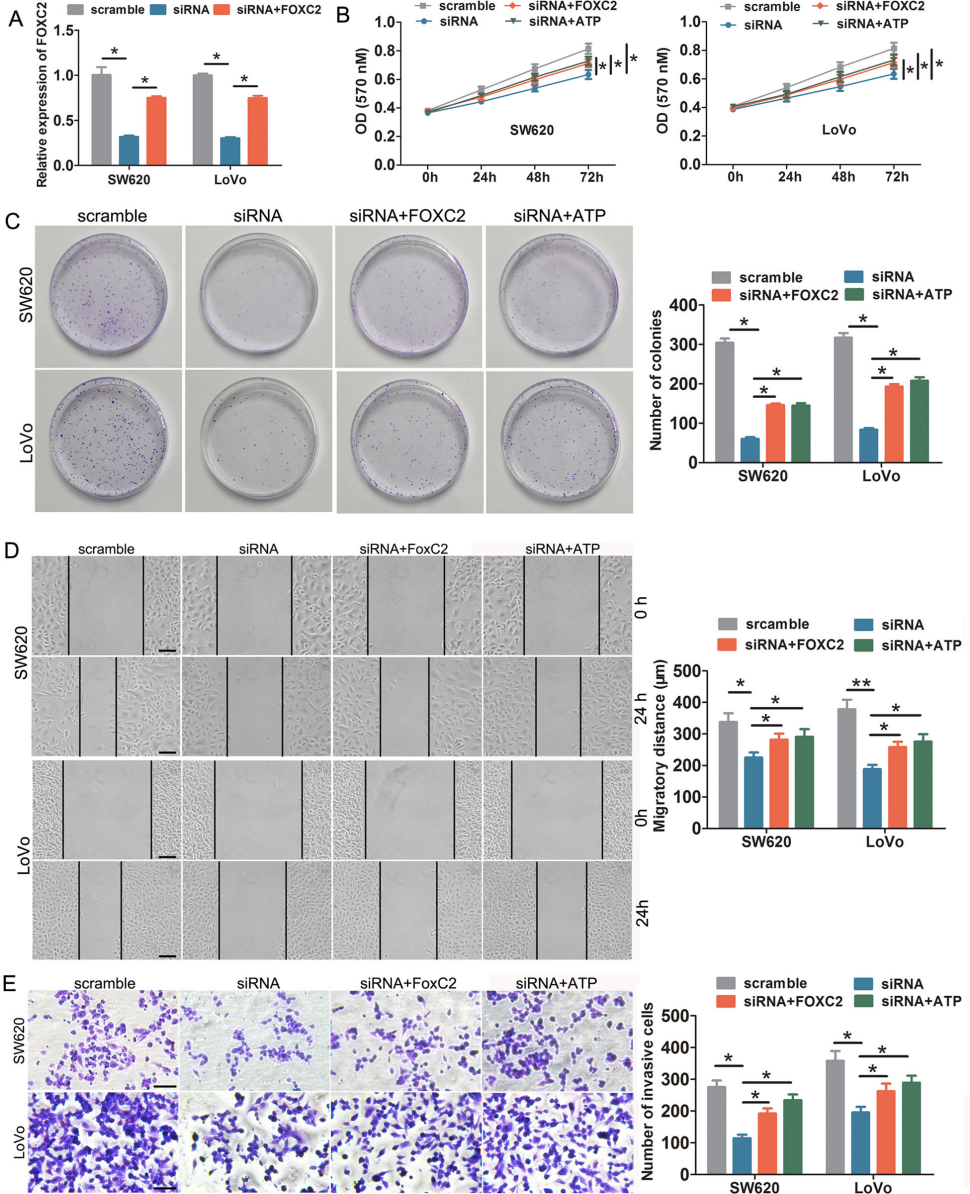
FOXC2-AS1 promoted cell proliferation, invasion, and metastasis in vitro and in vivo via FOXC2. **a** FOXC2 was elevated in FOXC2-AS1-depleting cells through transfecting FOXC2-overexpressed plasmids. MTT (**b**) and clone-formation assays (**c**) showed that ectopic expression of FOXC2 or ATP treatment could remarkably attenuate the inhibitory effects on cell proliferation and growth induced by FOXC2-AS1 depletion. Wound healing (**d**) and Transwell assay (**e**) showed that exogenously expressed FOXC2 or ATP treatment can obviously alleviate the impeded effects on CRC cell migration and invasion induced by FOXC2-AS1 depletion. Scale bars = 100 μm. *P < 0.05, **P < 0.01.

Previous reports have confirmed that FOXC2 could promote CRC progression^22–24^; therefore, we carried out rescue assays to assess whether FOXC2-AS1 promoted CRC cell growth, invasion, and metastasis via FOXC2. First, we elevated FOXC2 expression in FOXC2-AS1- depleting cells through transfection of FOXC2-overexpressed plasmids, and the expression of FOXC2 was verified by qRT-PCR (Fig. 5a). As shown in a series of functional experiments, ectopic expression of FOXC2 could obviously attenuate the inhibitory effects on cell proliferation, migration, and invasion in vitro mediated by FOXC2-AS1 knockdown (Fig. 5b–e). In in vivo subcutaneous xenograft experiments, FOXC2 obviously weakened the inhibitory effect on tumor volume, growth rates, and weight, causing by FOXC2-AS1 depletion (Fig. 2e–g). Moreover, FOXC2 overexpression could attenuate the liver metastatic potential of FOXC2-silencing LoVo cells in the metastasic model of mice (Fig. 3e, f). These results indicated that FOXC2-AS1 promoted CRC cell proliferation, invasion, and metastasis in vitro and in vivo via enhancing the stability of FOXC2 mRNA.

### FOXC2-AS1 promotes CRC progression via Ca^2+^-FAK signaling

**Fig. 6:**
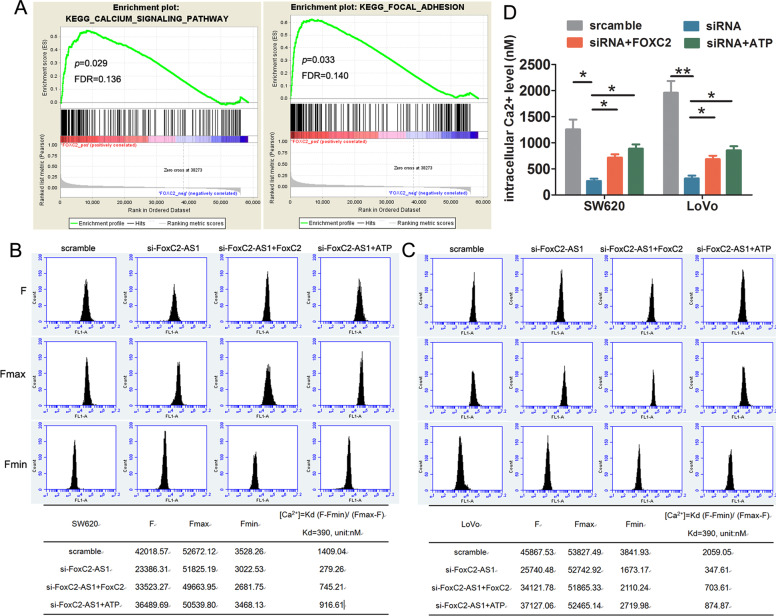
FOXC2-AS1 promotes CRC progression via regulating the intracellular Ca^2+^ level. **a** GESA analysis found that FOXC2 expression was positively correlated with calcium signaling pathway and focal adhesion. **b**, **c** FOXC2 expression or ATP treatment could mitigate the reduced intracellular Ca^2+^ level in FOXC2-AS1-silenced SW620 and LoVo cells; (**d**) data statistics of Ca^2+^ level. *P < 0.05, **P < 0.01.

To explore the molecular mechanisms regarding CRC progression mediated by FOXC2-AS1/FOXC2, GSEA of TCGA datasets was analyzed. We found that FOXC2 expression showed a positive correlation with calcium signaling pathway and focal adhesion (FA) (Fig. 6a). Therefore, we assessed the effect of FOXC2-AS1/FOXC2 expression on intracellular Ca^2+^ levels. The Ca^2+^ concentration was significantly reduced in FOXC2-AS1-silenced cells, but enhancing FOXC2 expression could mitigate the repressive effects. Moreover, when treated with the calcium ion mobilization agonist ATP, it also effectively alleviated the decreased Ca^2+^ level induced by FOXC2-AS1 knockdown (Fig. 6b–d). Remarkably, when treated with ATP, the inhibitory effects on cell growth, migration, and invasion caused by FOXC2-AS1 knockdown were also relieved (Fig. 5b–e), which implied that FOXC2-AS1/FOXC2 promotes CRC progression via regulation of the intracellular Ca^2+^ level.

**Fig. 7:**
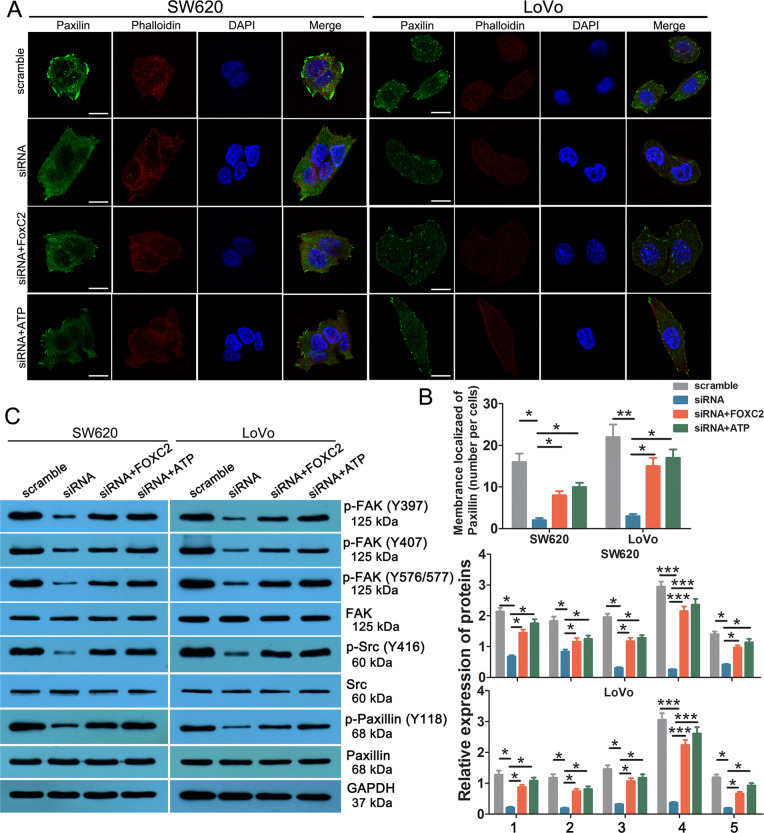
FOXC2-AS1 promotes CRC cell migration and invasion by facilitating FA through FAK signaling. **a** Focal adhesions were analyzed by co-localization of paxillin (green) and F actin (stained with phalloidin, red) in SW620 and LoVo cells. Scale bars = 50 μm. **b** Quantification of membrane-localized paxillin in each cell. (**c**) Western blot detected the expression of FAK signaling proteins in FOXC2-AS1-silenced, +FOXC2-overexpressed, and ATP-treated SW620 and LoVo cells. In all, 1 indicates the relative expression of p-FAK(Y394)/FAK, 2 indicates the relative expression of p-FAK(Y407)/FAK, 3 indicates the relative expression of p-FAK(Y576/577)/FAK, 4 indicates the relative expression of p-Src(Y394)/Src, and 5 indicates the relative expression of p-Paxillin(Y118)/Paxillin K. *P < 0.05, **P < 0.01, **P < 0.001.

Next, we investigated the effect of FOXC2-AS1/FOXC2 expression on cancer cell FA, a key subcellular structure that plays an important role in metastasis and invasion of tumors^25^. Immunofluorescence displayed that the number of FA was obviously reduced in FOXC2-AS1- silenced cells, but increasing FOXC2 expression could alleviate the inhibitory effects (Fig. 7a, b). As we know, focal adhesion kinase (FAK), Src, and paxillin are the principal components of the FA complex; the phosphorylation of these proteins was confirmed to be a critical process during the FA complex formation^25–27^. Western blot results are shown in Fig. 7c; the expression of phosphorylated FAK (Tyr397, Tyr407, and 575/577), Src (Tyr416), and paxillin (Tyr) was decreased in FOXC2-AS1-knockdown cells, while enhancing FOXC2 rescued its expression, which implied that FOXC2-AS1/FOXC2 contributes to CRC metastasis by promoting FA formation via FAK signaling. Notably, raising the intracellular Ca^2+^ level by ATP could effectively weaken the decreased numbers of FA, and rescued phosphorylated FAK, Src, and paxillin expression, which was caused by FOXC2-AS1 knockdown (Fig. 7c). It means that intracellular Ca^2+^ could affect FA formation and FAK signaling. Besides, we analyzed the expression of MMP-2 and MMP-9, two most widely studied members of MMP family, which were found to participate in ECM component degradation, cell migration, and invasion^28^. The result showed that the reduced expression of MMP-2 and MMP-9 mediated by FOXC2-AS1 silencing was significantly alleviated by FOXC2 overexpression or ATP treatment (Fig. S2). The above results reveal that lncRNA FOXC2-AS1 promotes CRC migration and invasion via activation of Ca^2+^-FAK signaling.

## Discussion

LncRNAs are emerging as shining stars in recent years; mounting evidences have verified lncRNAs playing a role in carcinogenesis and metastasis^8–11^. In this study, lncRNA FOXC2-AS1 was found to be upregulated in CRC specimens and cell lines. Most importantly, the expression in CRC with metastasis was obviously higher than in nonmetastatic tumor tissues, which was consistent with the data of GSE75050. Besides, the upregulated expression of FOXC2-AS1 was associated with lymphovascular invasion, TNM stage, distant metastasis, and poor survival in CRC patients. Furthermore, knockdown of FOXC2-AS1 significantly repressed CRC invasion and metastasis in in vitro and in vivo experiments. Thus, lncRNA FOXC2-AS1 was confirmed to be an oncogene that contributes to CRC metastasis in our data. For lncRNA, the subcellular localization largely determines its biological function. The cytoplasmic localized lncRNAs mainly participated in post-transcriptional regulation, while the nuclear-located lncRNAs exert epigenetic regulation, chromosomal interaction, and transcriptional regulation^13^. It is reported that FOXC2-AS1 acted as a ceRNA of miR-152 to promote the proliferation and progression of prostate cancer^14^. Another report found that FOXC2-AS1 contributed to tumorigenesis via epigenetic repression of p15 expression in NSCLC^15^. Here, we found that FOXC2-AS1 was mainly located in the nucleus base on FISH and RNA fraction analysis. It is implied that FOXC2-AS1 was involved in gene expression regulation.

It was reported that FOXC2-AS1 could enhance chemoresistance in osteosarcoma via promoting FOXC2 expression^17^, but the detailed mechanism was still unclear. Research has proposed that antisense RNA transcripts could regulate their sense genes at the transcriptional or post-transcriptional level^19^. FOXC2-AS1 is an antisense lncRNA that transcripts from the opposite strand of FOXC2. We observed that FOXC2-AS1 knockdown did not affect FOXC2 transcription, but influenced the stability of FOXC2 mRNA. Previous reports have confirmed that antisense lncRNAs may modulate sense mRNA stability via forming RNA duplexes^20,21^. Notably, we observed that exon 1 of FOXC2-AS1 was completely complementary with FOXC2, and we found that FOXC2-AS1 could utilize this mechanism to preserve RNA stability as FOXC2-AS1 could protect FOXC2 mRNA from RNase-mediated cleaving. Besides, we confirmed the direct interaction between FOXC2-AS1 and FOXC2 in CRC cells by RNA pull-down assay. Previous literature has reported that antisense lncRNA *Uchl1* is required for the association of overlapping sense protein-coding mRNA to active polysomes for translation^29^. However, whether this RNA duplex has an impact on mRNA translation was still uncertain. Perhaps, it has no impact on mRNA translation as FOXC2-AS1 could elevate FOXC2 protein expression, but this problem should be further explored. Previous studies have confirmed that FOXC2 is an oncogene and an independent prognostic factor in CRC, promoted by CRC progression^22–24^. In our functional assays, the inhibitory effect induced by FOXC2-AS1 silencing was mitigated by overexpression of FOXC2. But the underlying molecular mechanism requires further investigation.

Based on the GESA analysis in an expression-profiling data of FOXC2-overexpressing SW480 cells (GSE58980), we found that FOXC2 expression was positively associated with calcium signaling pathway and FA. As we know, Ca^2+^ is the most abundant intracellular second messenger, and is involved in a series of physiological functions through transduction of cellular signals; thus, it participates in a substantial diversity of cellular events, such as cell proliferation, differentiation, motility, apoptosis, and gene transcription^30^. The intracellular Ca^2+^ homeostasis is governed by a complex mechanism: the resting free cytosolic Ca^2+^ concentration is maintained at a very low level (10–100 nM). Once activated by cellular signals, the concentrations increase nearly 100 times either through release from inter-Ca^2+^ stores or through extracellular Ca^2+^ influx, thus generating Ca^2+^ signals that activate downstream effectors, such as calmodulin, calmodulin-dependent protein kinase II, and calpain (CAPN)^31^. Disruption of Ca^2+^ homeostasis facilitates the formation of malignant phenotypes^32^. Increasing evidence has demonstrated that Ca^2+^ plays an important role in tumor proliferation, invasion, and metastasis. For example, PLPP4 depletion suppresses lung carcinoma cell proliferation and tumorigenesis via impeding the influx of intracellular Ca^2+^^33^. Gong et al. reported that P2RX6 might function via ATP-induced Ca^2+^ influx to promote renal carcinoma cell migration and invasion^34^. ABAT suppressed Basl-like breast cancer by downregulation of intracellular Ca^2+^ concentration and inactivation of Ca^2+^-NFAT1 axis^35^. Here, we found that FOXC2-AS1 knockdown decreases the level of Ca^2+^ via suppression of FOXC2; moreover, elevating the level of Ca^2+^ could mitigate the oncogenic effect by FOXC2-AS1 silencing. Consistent with previous reports, our data suggested that FOXC2-AS1/FOXC2 promotes CRC progression by elevating Ca^2+^ levels.

Cell migration and invasion are a prerequisite for tumor metastasis. The migratory capacity facilitates cancer cells running away from the primary focus and disseminating through nearby circulations. The coordination of cell–substrate adhesion and detachment is necessary for cell migration^36^. FA is recognized dominant in this process, which acts through signal-linkage complexes between the extracellular matrix (ECM) and the cytoskeleton^37^. The FA assembly and disassembly, named FA turnover, determine cell motility, and the speed of FA dynamic cycle is considered to be the critical step in tumor invasion^38^. FAK is a well-known nonreceptor protein tyrosine kinase involved in FA dynamics^25^. FAK contains six tyrosine phosphorylation sites (Tyr397, 407, 576, 577, 861, and 925); FAK regulates cell adhesion and migration, mainly contributing to the autophosphorylation at Tyr397 site, which generates a high-affinity binding site for the SH2 domain of Src^39^. The interaction of FAK with Src subsequently phosphorylates Tyr407, 576, and 577, which lead to maximizing FAK activity^39^. Paxillin functions as a scaffolding protein that could recruit regulatory and structural proteins to regulate the dynamics of cell adhesion, modulating the turnover of FA complex and cytoskeletal remodeling, thus promoting cell migration and invasion^40^. When phosphorylating at Tyr118 by the FAK/Src complex, paxillin was activated; then multiple signaling pathways can coordinate to regulate cytoskeleton formation and promote cell migration^41^. In breast cancer, lung cancer, neuroblastoma, ovarian cancer, and hepatocellular carcinoma, studies have confirmed that FAK–Src–Paxillin cascade pathway modulates tumor cell proliferation, migration, and metastasis^26,42–45^. Our results proved that FOXC2 overexpression could reverse FOXC2-AS1 silencing-caused FA inhibition and FAK–Src–Paxillin cascade pathway inactivation. Notably, numerous evidence has demonstrated that Ca^2+^ signaling plays an important role in FA dynamics and FAK phosphorylation^46–48^. As shown in our results, elevating the level of Ca^2+^ mitigated FOXC2-AS1 silencing-induced FA inhibition and FAK–Src–Paxillin cascade pathway inactivation.

## Conclusions

In summary, our research demonstrated that lncRNA FOXC2-AS1 promotes CRC progression via activation of Ca^2+^/FAK signaling, and the new signal axis—Ca^2+^/FAK regulates cell FA and migration. These findings may provide a potentially effective therapeutic target for CRC progression.

## Materials and methods

### Clinical specimens

Sixty-eight cases of CRC specimens and 35 cases of adjacent tissues were diagnosed and collected in the second Xiangya hospital, Central South University between 2012 and 2016. No patient received chemotherapy and radiotherapy before surgery. The fresh specimens were snap-frozen in liquid nitrogen and then stored at −80 °C. All the patients were followed up at regular intervals after surgery. This research was approved by the Ethics Committee of The Second Xiangya Hospital of Central South University, and informed consent has been obtained by all the enrolled patients. The pathological information was obtained from patients’ medical records.

### Cell culture and transfection

CRC cell lines HT-29, HCT116, SW620, LoVo, and normal colonic epithelial cell line NCM460 were obtained from American Type Culture Collection (ATCC) and cultured following the manufacturer’s instructions.

FOXC2-AS1 siRNAs that directly target the sequence were synthesized by Invitrogen (Shanghai, China); scramble oligonucleotides were used as a negative control. The sequences of si-FOXC2-AS1 were as follows: si-FOXC2-AS1-1, 5′-GCGUGCCACUUAUUUCCAATA-3′; si-FOXC2-AS1-2, 5′-GCUGCGUAUUCGAUUCUCAGC-3′. FOXC2 cDNA ORF-overexpressed plasmid was purchased from Sino Biological (Beijing, China). Plasmids and siRNAs were transfected into cells with Lipofectamine 2000 (Thermo Fisher, USA) following the manufacturers’ instructions. Cells were collected 48 h after transfection.

### qRT-PCR assay

Total RNAs from tissues and cell lines were isolated with Trizol reagent (Thermo Fisher, USA). RNA reverse transcription was performed using GoScript^TM^ Kit (Promega, USA) according to the product’s protocol. qRT-PCR assay was conducted using SYBR Green I (TOYOBO, Japan) and performed on the LightCycler480 system (Roche, Germany). β-actin was applied as an internal control for normalizing. Each reaction was conducted in triplicate. The primer sequences were as follows: FOXC2-AS1 forward: 5′-CCTTCCTGGCTGTTCATCGG-3′, FOXC2-AS1 reverse: 5′-TGGAAATAAGTGGCACGCCC-3′; FOXC2 forward: 5′-CCTACCTGAGCGAGCAGAAT-3′, FOXC2 reverse: 5′-ACCTTGACGAAGCACTCGTT-3′; PDCD4-AS1 forward: 5′-CAGTCTAATGGGCAGAAGGGC-3′, PDCD4-AS1 reverse: 5′- AGGGGCACTGATCACATTCT-3′; β-actin forward: 5′-TTCCTTCCTGGGCATGGAGTC-3′, β-actin reverse: 5′-TCTTCATTGTGCTGGGTGCC-3′.

### MTT and clone-formation assays

For cell-proliferation examination, cells were plated into 96-well plates at the density of 5 × 10^3^ cells/well and cultured for 24 h. Next, in each well, we added 20 µl of MTT and incubated at 37 °C for 4 h. After removing the supernatant, each well was replenished with 150 µl of DMSO and incubated at 37 °C for 10 min. Finally, the absorbance was examined at 570 nm with a microplate reader (800TS, Biotek, USA). For the clone-formation experiment, 1 × 10^3^ cells were seeded into a 35-mm dish and cultured for 2 weeks. Then cell colonies were fixed in 4% paraformaldehyde (PFA) and stained with Giemsa dye.

### Cell migration and invasion assay

Cell migration was detected by wound-healing assay. Simply, cells were seeded in 12-well plates and grown to 100% confluence. Cell wounds were scratched with a 20-μl sterile pipette tip. Afterward, cells were washed with PBS 3 times and cultured with serum-free medium for 24 h. Wound closure was observed and measured the distance between the opposite edges of the wound.

Invasion assay was assessed with the BioCoat Matrigel Invasion chamber (Corning, USA). Cells (1 × 10^5^) in serum-free medium were transferred into the upper chamber coated with Matrigel, while the lower chambers were loaded with medium containing 20% FBS. After 48 h, the noninvading cells were wiped using cotton swabs. The invasive cells were fixed by 4% formaldehyde and stained in 0.1% crystal violet for 15 min. Finally, the images were captured under the microscope.

### Western blotting

Cells were lysed using RIPA buffer containing protease and phosphate inhibitors. Proteins were isolated from the supernatant of cell lysate. Equal amounts of proteins were separated using 10% SDS-PAGE gel and transferred to a polyvinylidene difluoride (PVDF) membrane (Thermo Fisher, USA). After blocking by 5% nonfat dry milk, the membrane was incubated with primary antibodies against FOXC2 (1:800, CST), p-FAK (Try397) (1:800, CST), p-FAK (Try407) (1:1000, Abcam), p-FAK (Try576/577) (1:1000, CST), FAK (1:1000, CST), p-Src (Try416) (1:1000, CST), Src (1:1000, CST), p-Paxillin (Try118) (1:500, Abcam), and Paxillin (1:1000, Abcam), overnight at 4 °C. After washing with PBS, the membrane was incubated with HRP-conjugated secondary antibody; the immune signals were examined using enhanced chemiluminescence reagent (Thermofisher, USA). GAPDH was used as the loading control.

### Subcellular fractionation

The nuclear and cytosolic fractions of LoVo and SW620 cells were isolated with PARIS Kit (Thermofisher, USA) following the manufacturer’s protocol.

### Fluorescence in situ hybridization (FISH)

The probe of FOXC2-AS1-1 was designed and synthesized by BersinBio (Guangzhou, China), and its sequences were 5′-GAGAAUCGAAUACGCAGCCGAUGAACAGCCAG-3′ conjugated with CY3. The slides of cells were fixed by 4% PFA for 20 min, and incubated with protein K for 30 min at 37 °C. After washing with PBS and denaturing at 73 °C for 3 min, the slides were incubated with the hybridization reaction solution that contained 5 ng/μl probes. The hybridization was carried out in a moist chamber at 42 °C overnight. Subsequently, the slides were rinsed with 25% deionized formamide/2 × saline sodium citrate (SSC) at 50 °C and a descending series of SSC at 50 °C. Finally, the slides were stained with DAPI. The signal was observed and photographed under fluorescent microscopy (Olympus, Japan).

Fresh CRC samples were immediately fixed with 4% paraformaldehyde (PFA) for 48 h and dehydrated by graded ethanol. After vitrification in dimethylbenzene and embedding in paraffin, the tissues were dewaxed and hydrated. The paraffin-embedded tissues were subjected to FISH referrred to the cell slides.

### Gene set enrichment analysis (GSEA)

The GSEA was launched to analyze gene sets correlated with FOXC2 in CRC. Gene expression data of CRC were downloaded from TCGA database. FOXC2 expression was set into high and low categories based on the median expression value. The FOXC2-correlated gene sets and pathways were explored in the c2.cp.kegg.v7.0.symbols.gmt data set by GSEA v3 soft. *P* < 0.05 and false-discovery rate (FDR) < 0.25 were the criterion for identifying statistically enriched genes.

### Intracellular Ca^2+^ measurement

Intracellular Ca^2+^ concentration was examined with Fluo-3 AM (Abcam, USA). Cells were seeded in 12-well plates and incubated with 2 μM Fluo-3 AM diluted in Krebs–Ringer buffer (Solarbio, Beijing, China) for 30 min. After removing the culture and washing with Krebs–Ringer buffer, the cells were collected and rinsed by PBS without Ca^2+^. Finally, the cells were measured by flow cytometry (Beckman Coulter, USA).

### Immunofluorescence

Cells (5 × 10^3^) were seeded in a 12-well plate paved with sterile slips. After 24 h, the cells were fixed, permeabilized, blocked, and then incubated with anti-paxillin antibody (ab32084, Abcam, USA) at a dilution of 1:150 overnight at 4 °C. The next day, the slips were incubated with fluorescently labeled secondary antibody and phalloidin (ab176756, Abcam, USA) for 2 h at room temperature. Subsequently, DAPI was used to stain nuclei. Finally, fluorescence was observed and imaged under the confocal laser-scanning microscope (LSM880, Zeiss, Germany).

### Nascent RNA capture analysis

Nascent RNA was isolated with Click-iT Nascent RNA Capture Kit (Life Technologies, USA) according to the product’s instructions. Next, qRT-PCR was conducted to detect the level of FOXC2 nascent RNA as mentioned above.

### RNA-stability analysis

Cells were treated with the transcriptional inhibitor actinomycin D (Sigma-Aldrich, USA) for 0 h, 1 h, 2 h, 4 h, 6 h, and 8 h, respectively. Cells were harvested at different time points, and RNA was isolated. Then, qRT-PCR was used to detect the level of FOXC2 mRNA as mentioned above.

### RNase A protection experiment

Cells were lysed in lysis buffer. Then the lysate was filtrated with a needle and stood on ice for 10 min. Next, the final solution was added with DNase I (Thermo Fisher) 12.5 U/ml and 125 mM NaCl, and divided into two parts. One part was treated with RNase A (200 ng/ml, Thermo Fisher), while the other was treated with RNase Inhibitor (250 U/ml, Thermo Fisher). After incubation at 37 °C for 30 min, the solutions were used for RNA isolation. qRT-PCR was used to detect the level of FOXC2-AS1 and FOXC2 as mentioned above.

### RNA pull-down analysis

FOXC2-AS1 and its antisense RNA were in vitro transcribed from the recombinant plasmids pCMV3-FOXC2-AS1 to produce biotin-labeled RNA with the Biotin RNA Labeling Mix (Roche, USA). Cell extracts were incubated with RNA probes, followed by streptavidin-mediated RNA pulldown. After RNA isolation, qRT-PCR was applied to investigate the interaction between FOXC2-AS1 and FOXC2.

### In vivo xenografts and metastasis assay

The animal experiments were conducted according to the protocols authorized by the Animal Care Committee of the second Xiangya Hospital, Central South University, and were raised with in vivo tumorigenicity. BALB/c nude mice (4–6-week old, male) were randomly divided into three groups (n = 5 for each group). In total, 1 × 10^7^ LoVo cells transfected with Lv-sh-FOXC2-AS1 (Genomeditecdh, Shanghai), Lv-sh-FOXC2-AS1 + FOXC2, or Lv-NC were injected subcutaneously into the mice right flank. Tumor growth was observed and recorded every 5 days. After 25 days, animals were killed after they were anesthetized and stripped the tumors. Tumor volume was calculated by the formula: (length × width^2^)/2.

For in vivo metastasis assays, Lv-sh-FOXC2-AS1, Lv-sh-FOXC2-AS1 + FOXC2, or control Lv-sh-NC lentivirus-transfected LoVo cells (5 × 10^6^/0.2 ml PBS) were intrasplenically injected into each mice (*n* = 6 for each group). All mice were euthanized 8 weeks later, and the liver was surgically excised. The liver tissues were fixed in formalin and embedded in paraffin for hematoxylin and eosin (HE) examination. Metastatic nodules were analyzed under microscopy.

### Statistical analysis

All the assays were executed at least three times, and the data were shown as mean ± standard deviation (SD). The statistical analysis was conducted by SPSS 18.0 software (SPSS Inc., IL, USA). Student’s t test was applied to analyze the differential expression between the two groups. A chi-square test was applied to assess the relationship between FOXC2-AS1 expression and clinicopathological characteristics of CRC. Survival analysis was estimated by the Kaplan–Meier method and evaluated using the log-rank test. *P* < 0.05 was identified as statistically significant.

## Data availability

All the data used and analyzed in this study are included in this article.

## Supplementary information

Figure S1

Figure S2

Supplementary figure legend
