# Supplementary figures and images for: LncRNA FOXC2-AS1 enhances FOXC2 mRNA stability to promote colorectal cancer progression via activation of Ca2+-FAK signal pathway
Source: Cell Death Dis. 2020 Jun 8;11(6):434. doi: 10.1038/s41419-020-2633-7 (PMC7280533; doi:10.1038/s41419-020-2633-7)

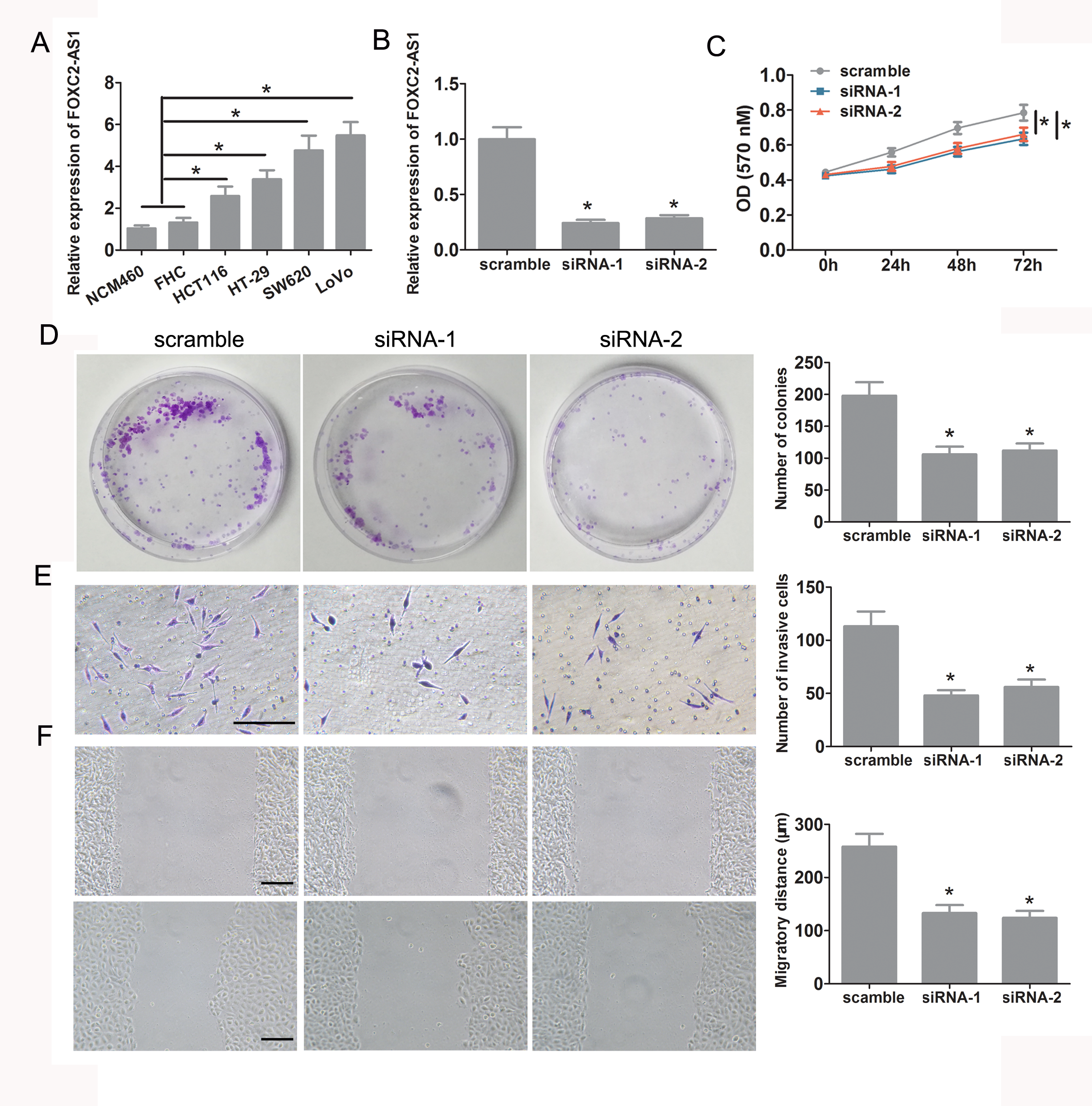

Supplement: Supplementary file 1 — Figure S1 [file 41419_2020_2633_MOESM1_ESM.tif]

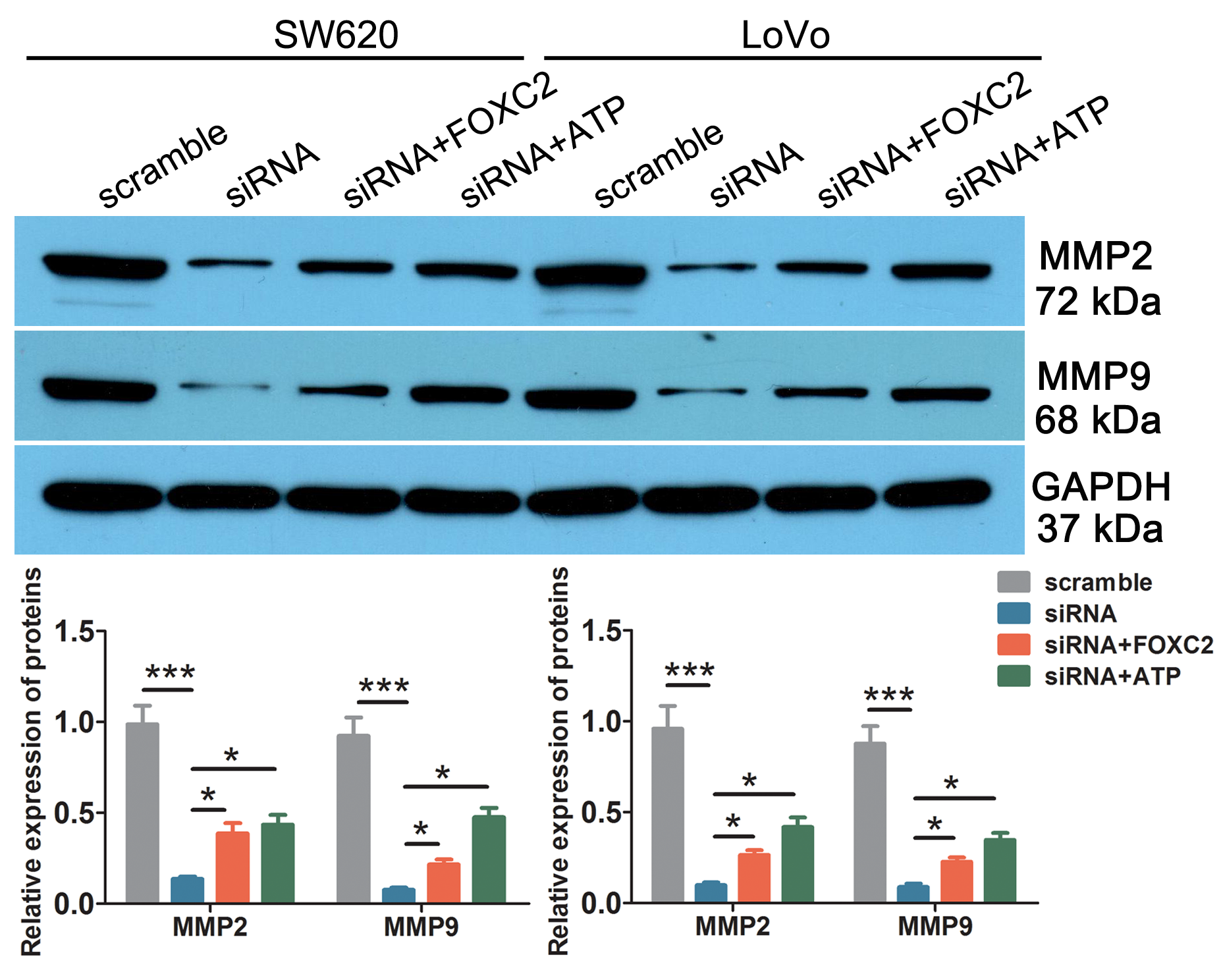

Supplement: Supplementary file 2 — Figure S2 [file 41419_2020_2633_MOESM2_ESM.tif]
