# Supplementary material for: LncRNA FOXC2-AS1 enhances FOXC2 mRNA stability to promote colorectal cancer progression via activation of Ca2+-FAK signal pathway
Source: Cell Death Dis. 2020 Jun 8;11(6):434. doi: 10.1038/s41419-020-2633-7 (PMC7280533; doi:10.1038/s41419-020-2633-7)
Supplement: Supplementary file 3 — Supplementary figure legend [file 41419_2020_2633_MOESM3_ESM.docx]

**Figure S1. Knockdown of FOXC2-AS1 suppresses normal colic cells FHC proliferation, migration and invasion.** (A) The endogenous expression of FOXC2-AS1 was examined in CRC cell lines (HCT116, HT-29, SW620, LoVo) and normal colonic cell lines(NCM460 and FHC). (B) Knockdown efficiency was examined by qRT-PCR in FHC cells. MTT (C) and clone formation assays (D) were used to examine the effect of FOXC2-AS1 depletion on FHC cell proliferation and growth. Wound healing (E) and Transwell assay (F) were performed to the effect of FOXC2-AS1 silencing on FHC cell migration and invasion. Scale bars=100 μm, *P<0.05.

**Figure S2. Western blot detected the expression of MMP2 and MMP9 proteins in FOXC2-AS1 silenced, FOXC2-AS1 silenced+FOXC2 overxpressed and ATP treated SW620 and LoVo cells**. *P<0.05, ***P<0.001.
